# Lifestyle habit change related to presenteeism change among Japanese employees

**DOI:** 10.3934/publichealth.2024037

**Published:** 2024-06-18

**Authors:** Momoko Tsuchida, Takafumi Monma, Sakiko Ozawa, Ayako Kikuchi, Fumi Takeda

**Affiliations:** 1 Graduate School of Comprehensive Human Sciences, University of Tsukuba, 1–1–1 Tennodai, Tsukuba-shi, Ibaraki 305–8577, Japan; 2 Institute of Health and Sport Sciences, University of Tsukuba, 1–1–1 Tennodai, Tsukuba-shi, Ibaraki 305–8577, Japan; 3 Research and Development Center for Lifestyle Innovation, University of Tsukuba, 1–2 Kasuga, Tsukuba-shi, Ibaraki 305–8550, Japan

**Keywords:** presenteeism, sleeping, corporate employee, lifestyle habits, sex difference

## Abstract

This study aimed to examine the relationship between changes in lifestyle habits and presenteeism change according to sex. This retrospective cohort study was conducted using data from health checkups, the World Health Organization Health and Work Performance Questionnaire (WHO-HPQ) short form, and health insurance claims for 9366 Japanese corporate employees in 2015 and 2016. Changes in 11 lifestyle habits of sleeping, eating, exercise, drinking, and smoking were classified into four patterns by combining lifestyle habits (good/poor): (a) no worsening, (b) worsening, (c) no improvement, and (d) improvement. A multiple regression analysis was conducted for each sex, with changes in the WHO-HPQ score as the objective variable, lifestyle habits change (worsening or improvement) as the explanatory variables, and age, job position, department, diseases, lifestyle habits, and WHO-HPQ score at baseline as adjustment variables. The results showed worsening of good lifestyle habits, such as sleeping, regular exercise, and frequency of drinking in men, while sleeping in women was associated with negative changes in the WHO-HPQ score. On the other hand, the improvement of poor lifestyle habit of sleeping was associated with positive changes in the WHO-HPQ score. These findings suggest that maintaining good lifestyle habits of sleeping for both sexes, and exercising and drinking for men, may be beneficial in maintaining work performance, while improving the poor lifestyle habit of sleeping for women may be beneficial in improving work performance.

## 1. Introduction

Maintaining and improving labor productivity has become essential in a society with a very low birth rate and an aging population in Japan [1]. Therefore, Japanese companies are required to improve their employees' work performance. The Ministry of Health, Labor, and Welfare (MHLW) promotes corporate productivity through health promotion, such as improving employee lifestyle habits [2], and requires employers to provide “specific health checkups and specific health guidance”, which involves health checkups focused on preventing metabolic syndrome and improving lifestyle habits. The specific health checkups include a biological examination and a questionnaire to measure the lifestyle habits of employees; those who are at high risk for lifestyle-related diseases are provided with active support to improve their lifestyle habits [3]. Moreover, the Ministry of Economy, Trade, and Industry (METI) promotes “health and productivity management (H&PM)”, with many companies aiming to improve productivity through employee health promotion [4].

In H&PM, employers are recommended to evaluate employees' work performance as presenteeism using a worldwide scale such as the World Health Organization Health and Work Performance Questionnaire (WHO-HPQ). While absenteeism is defined as an employee being absent from work due to health concerns, presenteeism is defined as “a decline in work performance due to the health problems of workers who are present at work” [5]. Since presenteeism accounts for approximately 64% of the total loss among health-related costs (absenteeism, presenteeism, and medical and pharmaceutical costs) [6], improving presenteeism is vital for companies. As part of H&PM, companies have implemented various initiatives focused on improving employees' lifestyle habits, such as diet (e.g., creating and supporting an environment in which employees can eat according to their dietary needs, providing applications, and other support to improve dietary habits) and exercise (e.g., encouraging exercise and providing tools to promote exercise, organizing sports events, and providing sports equipment outside the workplace) [7].

To implement effective measures to improve employee presenteeism, it is necessary to identify the key lifestyle habits that affect presenteeism; however, it remains unelucidated. Several cross-sectional studies found that smoking [8],[9], heavy alcohol consumption [8], insufficient fruit and vegetable intake [9], lack of exercise [10]–[12], and poor sleep [13],[14] were associated with presenteeism. Furthermore, our sex-based, cross-sectional study found that among 11 lifestyle habits (e.g., smoking, exercise, diet, drinking, and sleep), insufficient sleep, lack of regular exercise, and eating late evening meals were related to presenteeism in both sexes. In addition, slow walking speed, smoking, and skipping breakfast in men, while fast eating speed was associated with presenteeism in women were related to presenteeism [15].

However, there have been few longitudinal studies on the relationship between the changes in lifestyle habits and presenteeism change, and their findings are inconsistent. A study reported that among four lifestyle habits (smoking, heavy drinking, poor diet, and physical inactivity), only improvement in physical inactivity was associated with reduced presenteeism in United States corporate employees [16]. Another reported that among changes in Breslow's seven health habits (sleep, breakfast, snacking, physical exercise, alcohol drinking, smoking, and body mass index), only worsened sleep was associated with increased presenteeism in Japanese corporate employees [17]. Furthermore, there are sex differences in presenteeism [18], lifestyle habits [19],[20], and their relationship [15], suggesting that there may also be sex differences in the relationship between lifestyle habit change and presenteeism change.

Therefore, it is necessary to identify which of various lifestyle habits changes are associated with presenteeism changes in each sex This study aimed to examine the relationship between either the worsening or improvement of 11 comprehensive lifestyle habits, namely current smoking, regular exercise, physical activity, walking speed, eating late evening meals, eating snacks after dinner, skipping breakfast, eating speed, frequency of drinking, alcohol consumption per day, and sleeping obtained from specific health checkups and change of presenteeism by sex in Japanese employees. This will allow us to precisely identify interventions and target lifestyle habits that effectively maintain or improve work performance in each sex, based on real-world data from company employees. Considering previous findings [15]–[20], we hypothesized that (1) worsening of sleeping habits is associated with increased presenteeism, (2) improvement of exercise habits is associated with reduced presenteeism, and (3) there are sex differences in the relationship between lifestyle habit changes and presenteeism changes.

## 2. Materials and methods

### 2.1. Study design

A longitudinal study was conducted using anonymous data from specific health checkups, self-administered questionnaires on presenteeism, and health insurance claims of a Japanese company employees in 2015 and 2016. This data was provided under the non-disclosure agreement by the company and health insurance association of a private sector, and was approved for use in academic studies.

**Figure 1.**
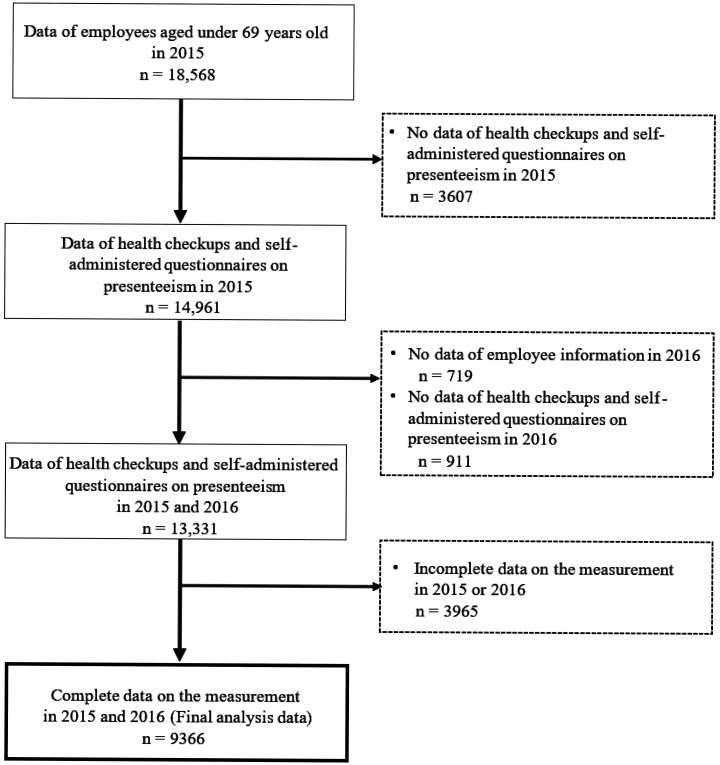
Flow chart of analysis data.

As shown in Figure 1, of 13,331 individuals aged under 69 years who belonged to the company and had data from specific health checkup and self-administered questionnaire on presenteeism in both 2015 and 2016, 9366 were included in the analysis, excluding 3965 with incomplete data on measurements described hereinafter. For the sensitivity analysis, 13,331 individuals were included by using multiple imputations to handle missing data.

#### 2.1.1. Ethics approval of research

This study was approved by the Research Ethics Committee of the Institute of Health and Sport Sciences of the University of Tsukuba, Japan (approval number: Tai 29–132).

### 2.2. Measures

#### 2.2.1. Attributes

Attributes including sex, age, job position (i.e., non-manager or manager), and department (i.e., sales, customer service, and administration) were obtained.

#### 2.2.2. Lifestyle habits

We used data obtained from self-administered questionnaires established in the standard Specific Health Check-up and Guidance program (2013 Edition) [3], which included the following 11 items: current smoking (“Do you smoke cigarettes regularly?”), regular exercise (“Are you in a habit of doing exercise to sweat lightly for over 30 minutes a time, 2 times weekly, for over a year?”), physical activity (“In your daily life do you walk or do any equivalent amount of physical activity for more than one hour a day?”), walking speed (“Is your walking speed faster than the speed of those of your age and sex?”), eating late evening meals (“Do you eat supper two hours before bedtime more than 3 times a week?”), eating snacks after dinner (“Do you eat snacks after dinner three days or more a week?”), skipping breakfast (“Do you skip breakfast more than 3 times a week?”), and sleeping (“Do you sleep well and enough?”). Each question was asked using a two-point rating scale representing either “yes” or “no.” Eating speed (“Is your eating speed quicker than others?”) was rated on a three-point scale as “quicker,” “normal,” or “slower”. Frequency of drinking (“How often do you drink?”) was also rated on a three-point scale as “everyday”, “sometimes”, or “rarely drink (cannot drink)”. Alcohol consumption per day (“How much do you drink per day?”) was rated on a four-point scale as “<180 mL/day”, “180–360 mL/day”, “360–540 mL/day”, or “≥540 mL/day”, where a glass (180 mL) of refined saké (rice wine) was equivalent to a medium bottle (500 mL) of beer, 110 mL of shochu (alcohol content, 25%), a glass (double, 60 mL) of whiskey, and two glasses (240 mL) of wine.

Of these, we dichotomized the items rated on either a three- or four-point scale as follows: eating speed as “quicker”, or “normal or slower”, frequency of drinking as “everyday” or “sometimes or rarely drink (cannot drink)”, and alcohol consumption per day as “light” (<360 mL/day for men; <180 mL/day for women) or “heavy” (≥360 mL/day for men; ≥180 mL/day for women) based on the criteria on the Second Phase of Health Japan 21 [21].

#### 2.2.3. Diseases

We evaluated eight diseases reported to be associated with presenteeism in prior studies (hypertension [22], dyslipidemia [22], diabetes [23], cancer [24], mental illness [25], infectious diseases [26], musculoskeletal diseases [27],[28], and oral diseases [29]). The assessed disease information, including related treatments, hospital procedures, drugs, and duration of primary disease, were obtained from health insurance claims data. We determined the presence of disease based on whether the individual received any treatments, procedures, or drugs for the primary disease, regardless of the duration.

The eight disease items corresponded to the ICD-10 (International Statistical Classification of Diseases and Related Health Problems 10th Revision) as follows: 1, hypertension (I10–I15); 2, dyslipidemia (E78); 3, diabetes (E10–E14); 4, cancer (stomach cancer [C16], colon cancer [C18–C20], lung/bronchial cancer [C33,C34], biliary/liver/pancreatic cancer [C22–C25], esophageal cancer [C15], oral/pharyngeal/laryngeal cancer [C00–C14 and C32], breast cancer [C50], and cervical cancer [C53]); 5, mental illness (mood (affective) disorders [F30–F39], neurotic, stress-related, and somatoform disorders [F40–F48], other mental and behavioral disorders (all of F00–F99 including nonorganic sleep disorders [F51], with excepting the above and dementias [F01,F03], mental and behavioral disorders due to psychoactive substance use [F10–F19], schizophrenia [F20–F29], and mental retardation [F70–F79]), and disorders of autonomic nervous system [G90]); 6, infectious diseases (intestinal infectious diseases [A00–A09], sequelae of infectious and parasitic diseases [B90–B94], acute upper respiratory infections [J00–J06], pneumonia [J12–J18], acute bronchitis [J20,J21]); 7, musculoskeletal diseases (M00–M99); and 8, oral diseases (diseases of the oral cavity, salivary glands, and jaw [K00–K14]).

#### 2.2.4. Presenteeism

We used the validated short-form Japanese version of the World Health Organization Health and Work Performance Questionnaire (WHO-HPQ) [30],[31]. The respondents were asked to answer the question “On a scale from 0 to 10, where 0 is the worst job performance anyone could have at your job and 10 is the performance of a top worker, how would you rate your overall job performance on the days you worked in the past 4 weeks (28 days)?” The final score was obtained by multiplying the respondents' answers by 10 (range: 0–100). A higher WHO-HPQ score indicated lower presenteeism, and thus, higher work performance.

### 2.3. Statistical analysis

First, the following were classified as poor lifestyle habits: “yes” for current smoking, “no” for regular exercise, “no” for physical activity, “no” for walking speed, “yes” for eating late evening meals, “yes” for eating snacks after dinner, “yes” for skipping breakfast, “quicker” for eating speed, “everyday” for frequency of drinking, “heavy (men: ≥360 mL/day, women: ≥180 mL/day)” for alcohol consumption per day, and “no” for sleeping.

Changes in lifestyle habits (“worsening” and “improvement”) were classified into four patterns by combining lifestyle habits (good/poor) in 2015 and 2016. First, of those habits that were “good” in 2015, those that were “good” in 2016 were defined as (a) “no worsening”; whereas those that were “poor” in 2016 were defined as (b) “worsening”. Second, of those habits that were “poor” in 2015, those that were “poor” in 2016 were defined as (c) “no improvement”; whereas those that were “good” in 2016 were defined as (d) “improvement”. The presenteeism change was defined as changes in the WHO-HPQ score by subtracting the WHO-HPQ score in 2015 from that in 2016.

After observing the basic statistics of all variables at baseline, the relationship between the worsening or improvement of lifestyle habits and changes in the WHO-HPQ score was analyzed using a t-test. Subsequently, multiple regression analyses were performed by sex, with changes in the WHO-HPQ score as the objective variable; the pattern of each lifestyle habit change as the explanatory variable; and age, job position, department, diseases (hypertension, dyslipidemia, diabetes, cancer, mental illness, infectious diseases, musculoskeletal diseases, and oral diseases), lifestyle habits, and WHO-HPQ score at baseline as the adjusted variables. During the analysis, the reference categories were “no worsening” and “no improvement”. Furthermore, in order to verify the robustness of the results, a sensitivity analysis was conducted using a data set with multiple imputation by chained equations to handle the missing values. An analysis of the imputed datasets reduces the potential bias introduced by missing data. This method assumes that data are missing at random, whereby any systematic differences between the missing and observed values could be explained by differences in the observed data [32]. The missing values were imputed according to a model that consisted of other all variables, and we used multiple imputations to create and analyze 20 multiply imputed datasets.

In all analyses, a p-value correction using the Benjamini–Hochberg method was conducted to consider type I errors. We ensured that there was no multicollinearity among variables. All data analyses were performed using the IBM SPSS Statistics for Windows (version 26.0; IBM Corp., Armonk, NY). The statistical significance was set at p < 0.05.

## 3. Results

The subject characteristics at baseline are presented in Table 1. The subjects of this study were 4899 men (52.3%) and 4467 women (47.7%), with a mean age of 43.1 ± 11.9 years (21–69 years). The mean WHO-HPQ scores of the subjects in 2015 and 2016 were 60.5 ± 17.1 and 61.1 ± 16.8, respectively.

Tables 2 and 3 show the changes in lifestyle habits and of the WHO-HPQ scores. The change in the WHO-HPQ score differed by worsening of sleeping in both men and women (Table 2) and improvement of sleeping only in women (Table 3).

The results of the multiple regression analysis adjusted for age, job position, department, diseases (hypertension, dyslipidemia, diabetes, cancer, mental illness, infectious diseases, musculoskeletal diseases, and oral diseases), lifestyle habits, and WHO-HPQ score at baseline are shown in Tables 4 and 5. Additionally, the results of the sensitivity analysis using a data set with the multiple imputation by chained equations to handle missing values are shown in Tables 4 and 5.

Regarding worsening of lifestyle habits (Table 4), for men, worsening of regular exercise (β = −0.058, p = 0.024), frequency of drinking (β = −0.044, p = 0.009), and sleeping (β = −0.063, p < 0.001) were associated with negative changes in the WHO-HPQ score. For women, skipping breakfast (β = −0.034, p = 0.018) and sleeping (β = −0.048, p = 0.003) were associated with negative changes in the WHO-HPQ score. Moreover, the sensitivity analysis showed similar results, except that for women, worsening of skipping breakfast was not associated with change in the WHO-HPQ score.

Regarding improvement of lifestyle habits (Table 5), for men, no variables were associated with changes in the WHO-HPQ score. For women, improvement of sleeping (β = 0.051, p = 0.013) was associated with positive changes in the WHO-HPQ score. However, the sensitivity analysis showed that improvement of sleeping for men and of regular exercise and sleeping for women were associated with positive changes in the WHO-HPQ score.

**Table 1.** Subject characteristics.

		All (n = 9366)	
		n (%) or Mean ± SD	
**Attributes**			
Sex	Men	4899	(52.3)
	Women	4467	(47.7)
Age		43.1	±11.9
	21–29 y	1736	(18.5)
	30–39 y	1897	(20.3)
	40–49 y	2560	(27.3)
	50–59 y	2372	(25.3)
	60–69 y	801	(8.6)
Department	Sales	4831	(51.5)
	Customer service	3405	(36.4)
	Administration	1130	(12.1)
Job position	Non-manager	7700	(82.2)
	Manager	1666	(17.8)
**Lifestyle habits**			
Current smoking (Yes)		1611	(17.2)
Regular exercise (No)		7690	(82.1)
Physical activity (No)		7285	(77.8)
Walking speed (No)		5386	(57.5)
Eating late evening meals (Yes)		3372	(36.0)
Eating snacks after dinner (Yes)		1152	(12.3)
Skipping breakfast (Yes)		2077	(22.2)
Eating speed (quicker)		3476	(37.1)
Frequency of drinking (Everyday)		2940	(31.4)
Alcohol consumption per day (Heavy)		3826	(40.8)
Sleeping (No)		3180	(34.0)
**Diseases**			
Hypertension		1120	(12.0)
Dyslipidemia		1378	(14.7)
Diabetes		569	(6.1)
Cancer		241	(2.6)
Mental illness		610	(6.5)
Infectious diseases		5062	(54.0)
Musculoskeletal diseases		2309	(24.7)
Oral diseases		4735	(50.6)
**WHO-HPQ score**		60.5	±17.1

Note: SD: standard deviation; WHO-HPQ: World Health Organization Health and Work Performance Questionnaire.

**Table 2.** Worsening of lifestyle habits and changes in the WHO-HPQ score.

		Men				Women			
	Worsening	n	(%)	Mean ± SD	P-value*	n	(%)	Mean ± SD	P-value*
Current smoking	No	3503	(97.8)	0.7 ± 15.9	0.091	4144	(99.3)	0.8 ± 18.2	1.000
	Yes	80	(2.2)	−2.4 ± 17.9		28	(0.7)	−0.7 ± 14.1	
Regular exercise	No	923	(75.7)	1.5 ± 15.5	0.132	311	(68.1)	0.0 ± 17.2	0.617
	Yes	296	(24.3)	−0.2 ± 15.6		146	(31.9)	−1.4 ± 18.5	
Physical activity	No	778	(64.1)	0.4 ± 16.1	1.000	529	(61.0)	0.8 ± 18.2	1.000
	Yes	436	(35.9)	0.5 ± 15.9		338	(39.0)	0.9 ± 17.7	
Walking speed	No	1977	(81.3)	0.7 ± 15.6	1.000	1224	(79.0)	0.4 ± 17.2	0.836
	Yes	454	(18.7)	0.6 ± 16.7		325	(21.0)	1.1 ± 19.7	
Eating late evening meals	No	2241	(80.6)	1.2 ± 15.1	0.289	2786	(86.7)	0.8 ± 17.6	1.000
	Yes	539	(19.4)	0.1 ± 17.3		428	(13.3)	0.6 ± 19.4	
Eating snacks after dinner	No	4216	(94.4)	0.7 ± 16.1	1.000	3472	(92.7)	0.6 ± 18.2	1.000
	Yes	251	(5.6)	0.9 ± 16.1		275	(7.3)	1.0 ± 18.1	
Skipping breakfast	No	3592	(95.1)	1.0 ± 15.6	0.402	3301	(94.0)	0.6 ± 17.7	0.497
	Yes	184	(4.9)	−0.5 ± 17.1		212	(6.0)	−0.6±19.8	
Eating speed	No	2443	(87.5)	0.5 ± 16.1	1.000	2865	(92.4)	1.0 ± 18.0	1.000
	Yes	348	(12.5)	0.7 ± 16.7		234	(7.6)	0.3 ± 18.1	
Frequency of drinking	No	2371	(89.5)	0.6 ± 16.3	0.165	3614	(95.7)	0.8 ± 18.0	1.000
	Yes	277	(10.5)	−1.2 ± 18.3		164	(4.3)	1.4 ± 18.3	
Alcohol consumption per day	No	2722	(87.3)	1.3 ± 15.9	0.671	2044	(84.4)	0.6 ± 17.7	0.278
	Yes	395	(12.7)	0.4 ± 17.4		379	(15.6)	−0.5 ± 18.7	
Sleeping	No	2888	(84.5)	1.1 ± 15.2	0.014	2267	(81.9)	1.0 ± 17.7	0.036
	Yes	531	(15.5)	−1.0 ± 18.5		500	(18.1)	−0.9 ± 17.9	

Note: SD: standard deviation; WHO-HPQ: World Health Organization Health and Work Performance Questionnaire; t-test; *P-value correction using the Benjamini–Hochberg method was conducted.

**Table 3.** Improvement of lifestyle habits and changes in the WHO-HPQ score.

		Men				Women			
	Improvement	n	(%)	Mean ± SD	P-value*	n	(%)	Mean ± SD	P-value*
Current smoking	No	1222	(92.9)	0.9 ± 16.9	0.111	265	(89.8)	−0.5±18.4	1.000
	Yes	94	(7.1)	−2.0 ± 14.5		30	(10.2)	−1.3±18.7	
Regular exercise	No	3326	(90.4)	0.6 ± 16.4	0.953	3838	(95.7)	0.7±18.3	0.084
	Yes	354	(9.6)	0.0 ± 15.9		172	(4.3)	3.2±17.2	
Physical activity	No	3200	(86.8)	0.9 ± 16.3	0.085	3219	(89.4)	0.6±18.2	1.000
	Yes	485	(13.2)	−0.4 ± 15.2		381	(10.6)	1.0±18.5	
Walking speed	No	2062	(83.5)	0.7 ± 16.5	1.000	2620	(89.8)	0.7±18.1	1.000
	Yes	406	(16.5)	0.4 ± 16.2		298	(10.2)	0.7±20.9	
Eating late evening meals	No	1622	(76.5)	0.0 ± 16.8	0.362	859	(68.6)	0.0±19.0	1.000
	Yes	497	(23.5)	1.0 ± 17.1		394	(31.4)	0.9±18.9	
Eating snacks after dinner	No	207	(47.9)	0.3 ± 17.6	1.000	398	(55.3)	−0.2±18.4	0.106
	Yes	225	(52.1)	−0.2 ± 14.6		322	(44.7)	2.1±17.9	
Skipping breakfast	No	916	(81.6)	−0.3 ± 17.8	0.317	761	(79.8)	1.0±19.0	1.000
	Yes	207	(18.4)	1.2 ± 16.4		193	(20.2)	1.7±20.5	
Eating speed	No	1783	(84.6)	1.0 ± 16.1	1.000	1088	(79.5)	−0.2±18.6	0.431
	Yes	325	(15.4)	0.6 ± 16.5		280	(20.5)	1.2±18.3	
Frequency of drinking	No	2003	(89.0)	1.1 ± 15.6	0.693	552	(80.1)	−0.8±18.6	0.177
	Yes	248	(11.0)	0.3 ± 16.5		137	(19.9)	1.9±19.1	
Alcohol consumption per day	No	1277	(71.7)	−0.1 ± 16.3	1.000	1568	(76.7)	0.7±18.5	0.766
	Yes	505	(28.3)	−0.4 ± 15.8		476	(23.3)	1.5±18.4	
Sleeping	No	1031	(69.7)	0.7 ± 17.0	1.000	1272	(74.8)	0.0±19.0	0.015
	Yes	449	(30.3)	0.2 ± 17.2		428	(25.2)	2.6±18.2	

Note: SD: standard deviation; WHO-HPQ: World Health Organization Health and Work Performance Questionnaire; t-test; *P-value correction using the Benjamini–Hochberg method was conducted.

**Table 4.** Relationship between worsening of lifestyle habits and changes in the WHO-HPQ score.

**Men**	Multiple regression analysis						Sensitivity analysis			
	n*	B	95% CI	β	P-value**	Adjusted R^2^	n*	B	SE	P-value**
Current smoking	3583	−2.807	(−5.82 to 0.21)	−0.026	0.093	0.281	4541	−2.338	1.403	0.132
Regular exercise	1219	−2.082	(−3.83 to −0.34)	−0.058	0.024	0.288	1522	−1.911	0.812	0.023
Physical activity	1214	−0.351	(−1.96 to 1.26)	−0.010	1.000	0.304	1482	−0.584	0.742	0.948
Walking speed	2431	−0.578	(−1.95 to 0.80)	−0.014	0.901	0.293	2886	−0.420	0.633	1.000
Eating late evening meals	2780	−0.931	(−2.22 to 0.36)	−0.024	0.247	0.281	3616	−0.873	0.575	0.202
Eating snacks after dinner	4467	−0.026	(−1.78 to 1.73)	0.000	1.000	0.285	5566	−0.278	0.799	1.000
Skipping breakfast	3776	−1.058	(−3.07 to 0.95)	−0.015	0.555	0.277	4682	−0.733	0.915	0.775
Eating speed	2791	−0.045	(−1.60 to 1.51)	−0.001	1.000	0.282	3289	0.295	0.744	1.000
Frequency of drinking	2648	−2.372	(−4.13 to −0.62)	−0.044	0.009	0.286	3623	−2.359	0.816	0.004
Alcohol consumption per day	3117	−0.585	(−2.04 to 0.87)	−0.012	1.000	0.290	3570	−0.375	0.710	1.000
Sleeping	3419	−2.722	(−3.96 to −1.49)	−0.063	<0.001	0.307	4202	−3.262	0.559	<0.001

**Women**	Multiple regression analysis						Sensitivity analysis			
	n*	B	95% CI	β	P-value**	Adjusted R^2^	n*	B	SE	P-value**

Current smoking	4172	−0.329	(−5.97 to 5.31)	−0.001	1.000	0.310	6586	0.565	2.468	1.000
Regular exercise	457	−2.068	(−5.19 to 1.05)	−0.055	0.354	0.263	707	−1.128	1.239	0.665
Physical activity	867	−0.045	(−2.10 to 2.01)	−0.001	1.000	0.326	1243	−1.119	0.873	0.275
Walking speed	1549	−0.775	(−2.65 to 1.10)	−0.018	1.000	0.284	2230	−0.381	0.789	1.000
Eating late evening meals	3214	−1.526	(−3.04 to −0.01)	−0.029	0.059	0.328	5062	−1.163	0.621	0.067
Eating snacks after dinner	3747	−1.005	(−2.87 to 0.86)	−0.014	0.639	0.311	5773	−0.550	0.763	1.000
Skipping breakfast	3513	−2.554	(−4.64 to −0.47)	−0.034	0.018	0.306	5351	−1.527	0.839	0.084
Eating speed	3099	−1.498	(−3.50 to 0.51)	−0.022	0.196	0.306	4557	−0.492	0.845	1.000
Frequency of drinking	3778	0.526	(−1.84 to 2.89)	0.006	1.000	0.311	6119	0.102	1.099	1.000
Alcohol consumption per day	2423	−1.147	(−2.82 to 0.52)	−0.023	0.279	0.305	2909	−0.781	0.803	0.520
Sleeping	2767	−2.219	(−3.67 to −0.77)	−0.048	0.003	0.305	4121	−1.414	0.596	0.018

Note: WHO-HPQ: World Health Organization Health and Work Performance Questionnaire; *The number of subjects who had “good” habits in each lifestyle habits at baseline; **P-value correction using the Benjamini–Hochberg method was conducted; Adjusted variables: age, job position, department, diseases (hypertension, dyslipidemia, diabetes, cancer, mental illness, infectious diseases, musculoskeletal diseases, and oral diseases), lifestyle habits and WHO-HPQ score at baseline; Code of independent variables: No worsening, 0; worsening, 1.

**Table 5.** Relationship between improvement of lifestyle habits and changes in the WHO-HPQ score.

**Men**	Multiple regression analysis						Sensitivity analysis			
	n*	B	95% CI	β	P-value**	Adjusted R^2^	n*	B	SE	P-value**
Current smoking	1316	−1.143	(−4.17 to 1.88)	−0.018	0.721	0.279	1637	−1.505	1.364	0.371
Regular exercise	3680	0.235	(−1.30 to 1.77)	0.004	1.000	0.285	4597	0.607	0.698	0.604
Physical activity	3685	−0.604	(−1.93 to 0.72)	−0.013	0.510	0.277	4612	−0.019	0.604	1.000
Walking speed	2468	0.438	(−1.08 to 1.95)	0.010	1.000	0.275	3226	0.860	0.691	0.260
Eating late evening meals	2119	0.672	(−0.78 to 2.12)	0.017	0.443	0.289	2508	0.434	0.669	0.946
Eating snacks after dinner	432	0.374	(−2.32 to 3.07)	0.012	1.000	0.263	555	0.583	1.202	1.000
Skipping breakfast	1123	1.472	(−0.78 to 3.72)	0.033	0.220	0.296	1389	1.731	1.034	0.104
Eating speed	2108	0.180	(−1.45 to 1.81)	0.004	1.000	0.284	2563	−0.088	0.764	1.000
Frequency of drinking	2251	−0.121	(−1.91 to 1.67)	−0.002	1.000	0.277	2509	0.028	0.853	1.000
Alcohol consumption per day	1782	−0.216	(−1.69 to 1.26)	−0.006	1.000	0.265	2002	0.066	0.702	1.000
Sleeping	1480	1.305	(−0.36 to 2.97)	0.035	0.125	0.247	1925	1.692	0.751	0.024

**Women**	Multiple regression analysis						Sensitivity analysis			
	n*	B	95% CI	β	P-value**	Adjusted R^2^	n*	B	SE	P-value**

Current smoking	295	−2.581	(−8.82 to 3.66)	−0.042	0.763	0.317	413	−2.191	2.713	0.769
Regular exercise	4010	2.382	(0.04 to 4.73)	0.026	0.051	0.315	6101	2.140	0.974	0.031
Physical activity	3600	−0.483	(−2.12 to 1.15)	−0.008	1.000	0.306	5463	0.085	0.687	1.000
Walking speed	2918	−0.022	(−1.85 to 1.80)	0.000	1.000	0.321	4568	0.888	0.744	0.320
Eating late evening meals	1253	1.174	(−0.82 to 3.17)	0.029	0.305	0.262	1749	1.461	0.846	0.103
Eating snacks after dinner	720	1.220	(−1.10 to 3.54)	0.033	0.416	0.294	1036	0.595	0.986	1.000
Skipping breakfast	954	0.474	(−2.11 to 3.06)	0.010	1.000	0.311	1413	0.506	1.054	1.000
Eating speed	1368	1.023	(−1.04 to 3.09)	0.022	0.522	0.306	1982	1.035	0.924	0.413
Frequency of drinking	689	0.770	(−2.34 to 3.88)	0.016	1.000	0.277	768	−0.034	1.525	1.000
Alcohol consumption per day	2044	0.171	(−1.44 to 1.78)	0.004	1.000	0.312	2348	0.160	0.789	1.000
Sleeping	1700	2.225	(0.47 to 3.98)	0.051	0.013	0.313	2688	1.944	0.722	0.007

Note: WHO-HPQ, World Health Organization Health and Work Performance Questionnaire; *The number of subjects who had “poor” habits in each lifestyle habits at baseline; **P value correction using the Benjamini–Hochberg method was conducted; Adjusted variables: age, job position, department, diseases (hypertension, dyslipidemia, diabetes, cancer, mental illness, infectious diseases, musculoskeletal diseases, and oral diseases), lifestyle habits and WHO-HPQ score at baseline; Code of independent variables: No improvement, 0; improvement, 1.

## 4. Discussion

This study examined the relationship between worsening or improvement of 11 lifestyle habits (current smoking, regular exercise, physical activity, walking speed, eating late evening meals, eating snacks after dinner, skipping breakfast, eating speed, frequency of drinking, alcohol consumption per day, and sleeping) and presenteeism change over 1 year by sex. Since the WHO-HPQ score of the study subjects at baseline (60.5 ± 17.1) was similar to that of Japanese workers aged 20 to 60 years (60.5 ± 17.8) [33], the presenteeism in this study population may be generalized to Japanese workers of the same age group.

The key results of this study, which were common across multiple regression analyses and sensitivity analyses, are as follows. First, worsening of sleeping for both sexes, and of regular exercise and frequency of drinking for men, were associated with increased presenteeism. Second, improvement of sleeping for women was associated with reduced presenteeism.

First, regarding worsening of lifestyle habits, the results of both multiple regression analyses and sensitivity analyses showed that worsening of sleeping was associated with increased presenteeism in both sexes, thus this association is considered to be robust. This is consistent with previous findings [17] and supports the first hypothesis (i.e., that worsening of sleeping habits is associated with increased presenteeism). Our finding suggests that to prevent the decline in employees' work performance, maintaining sufficient sleep is vital, regardless of sex.

However, other lifestyle habits associated with increased presenteeism differed by sex. For men, worsening of regular exercise and frequency of drinking were associated with increased presenteeism in the results of both multiple regression analyses and sensitivity analyses; thus, these associations are considered to be robust. The former is similar to our cross-sectional study findings that the exercise habits were associated with presenteeism, especially among men [15], whereas the latter is not. This indicates that while there is no relationship between alcohol consumption and presenteeism at the cross-sectional time point, an increased frequency of alcohol consumption leads to increased presenteeism in men. In a national survey in Japan, men drank more frequently than women [19], and the proportion of those who worsened the frequency of drinking was also higher than that of women in our study. In addition, the risk of alcohol-related presenteeism was higher among men [34]. This may be because that the worsening frequency of drinking was associated with increased presenteeism only for men and suggests that it is important for male employees to avoid increasing their drinking frequency to maintain their work performance.

On the other hand, for women, as the result of the multiple regression analyses, the worsening of skipping breakfast was associated with increased presenteeism. Skipping breakfast is known to decrease intellectual work capacity [35] and be a factor of dysmenorrhea or menstrual cramps [36],[37], which are associated with presenteeism in women [38]. Thus, it is possible that the worsening of skipping breakfast was associated with increased presenteeism in women. However, this association was not observed in the result of the sensitivity analysis, which is consistent with a previous study that found no relationship between the worsening of dietary habits and presenteeism change in Japanese employees [17]. Therefore, further validation studies are needed in this regard.

Second, regarding improvement of lifestyle habits, the results of both multiple regression analyses and sensitivity analyses showed that improved sleeping was associated with reduced presenteeism only for women; thus, this association is considered to be robust. On the other hand, only the result of the sensitivity analysis showed that improvement of sleeping for men, and of regular exercise and sleeping for women, were also associated with reduced presenteeism. The former suggested that changes in sleeping may be key to changes in work performance for both sexes, and the latter is consistent with a previous study of U.S. corporate employees, which reported that improvement of physical inactivity was related to reduced presenteeism [16] and supported the second hypothesis (i.e., that improvement of exercise habits is associated with reduced presenteeism). However, these need to be explored through further validation studies and other population surveys.

Our previous cross-sectional study that addressed 11 lifestyle habits showed that insufficient sleep, lack of regular exercise, and eating late evening meals in both sexes, slow walking speed, smoking, and skipping breakfast in men, and fast eating speed in women were related to presenteeism [15]. However, a more detailed examination in this longitudinal study provided new evidence that the relationship between changes in these lifestyle habits and presenteeism change reflected worsening rather than improvement, especially among men; this suggests that worsening of lifestyle habits is particularly likely to lead to increased presenteeism. Therefore, to prevent a decline in work performance, it should be vital to preserve sufficient sleep in both sexes, and regular exercise and a low frequency of drinking for men. On the other hand, to improve work performance, women's lack of sleep must be improved.

Currently, Japanese companies provide health guidance to employees under the Specific Health Guidance to improve poor lifestyle habits for those at high risk of developing lifestyle-related diseases [3]. Moreover, companies have developed initiatives to improve dietary habits through environmental improvement and app provision, as well as to improve exercise habits through the organization of sporting events and partnerships with sports clubs outside the workplace under “H&PM” [4]. Although these are behavior change initiatives targeting individuals with poor lifestyle habits, our findings suggest the importance of maintaining good habits of sleeping, exercise, and drinking as a measures against presenteeism. Therefore, in addition to lifestyle modification efforts targeting individuals, the development of a comfortable psychosocial work environment is considered important. This is because workplace environmental factors such as long working hours and occupational stress are related to sleep quality [39],[40], exercise habits [41], and drinking habits [42]. Companies should provide an appropriate work environment, including adequate working hours and workloads, so that employees can maintain healthy lifestyles.

This study had several limitations. First, since the study participants were employees of a Japanese company, our study had a selection bias. In addition, the magnitude of associations between lifestyle habits and presenteeism were small and the results of the sensitivity analyses showed some different from the main results. Therefore, the generalization of our study findings to the entire Japanese employee populations was difficult, and more precise validation studies and further investigations in other populations are warranted. Second, because this study was conducted over 1 year, the long-term effects of lifestyle improvement and continuity, and the causal relationship between lifestyle habits changes and changes in presenteeism are unknown. Therefore, it is necessary to carefully determine the causal relationship between lifestyle habits changes and changes in presenteeism through long-term follow-ups. Finally, because the data on lifestyle habits in this study were collected using questionnaires from specific health examination checkups, reporting biases could not be ruled out. Further verification using a wider range of objectives and survey data is necessary.

Despite the above limitations, to the best of our knowledge, this is the first study to examine the relationship between changes in both directions (worsening or improvement) of multiple lifestyle habits and presenteeism change by sex using 11 lifestyle items obtained from the specific health checkup. The results of this study suggested that for maintaining work performance, preserving sufficient sleep in both sexes, and regular exercise and frequency of drinking in men are critical. On the other hand, to improve work performance, improving sleeping in women is essential. This indicates that the target populations, as well as effective interventions of lifestyle habits, excluding sleeping, to either maintain or improve work performance, differ by sex. The findings of this study may be useful in considering lifestyle interventions for employees to maintain and improve work performance.

## 5. Conclusions

This study examined the relationship between changes in lifestyle habits (worsening/improvement) and presenteeism change among Japanese employees by sex. The results showed that worsening of sleeping, regular exercise, and frequency of drinking for men, while worsening of sleeping for women, were associated with increased presenteeism. On the other hand, no improvement in lifestyle habits for men, and improvement of sleeping for women, were associated with reduced presenteeism. These findings suggest that maintaining good lifestyle habits of sleeping for both sexes, and exercise and drinking for men, may be beneficial in maintaining work performance, while improving poor sleeping may be beneficial for women to improve work performance. Therefore, a supportive work environment should be enhanced to help employees maintain good lifestyle habits. However, further validation studies and investigations in other populations are needed.

## Use of AI tools declaration

The authors declare they have not used Artificial Intelligence (AI) tools in the creation of this article.
